# FOXA1 levels are decreased in pleural breast cancer metastases after adjuvant endocrine therapy, and this is associated with poor outcome

**DOI:** 10.1002/1878-0261.12353

**Published:** 2018-10-12

**Authors:** Willemijne Schrijver, Karianne Schuurman, Annelot van Rossum, Marjolein Droog, Carmen Jeronimo, Sofia Salta, Rui Henrique, Jelle Wesseling, Cathy Moelans, Sabine C. Linn, Michel van den Heuvel, Paul van Diest, Wilbert Zwart

**Affiliations:** ^1^ Department of Pathology University Medical Center Utrecht The Netherlands; ^2^ Division of Oncogenomics Oncode Institute The Netherlands Cancer Institute Amsterdam The Netherlands; ^3^ Division of Molecular Pathology The Netherlands Cancer Institute Amsterdam The Netherlands; ^4^ Cancer Biology and Epigenetics Group Research Center (CI‐IPOP) Portuguese Oncology Institute of Porto Portugal; ^5^ Department of Pathology Portuguese Oncology Institute of Porto (IPO Porto) Portugal; ^6^ Division of Pathology The Netherlands Cancer Institute Amsterdam The Netherlands; ^7^ Division of Medical Oncology The Netherlands Cancer Institute Amsterdam The Netherlands; ^8^ Division of Thoracic Oncology The Netherlands Cancer Institute Amsterdam The Netherlands; ^9^ Laboratory of Chemical Biology and Institute for Complex Molecular Systems Department of Biomedical Engineering Eindhoven University of Technology The Netherlands

**Keywords:** acquired endocrine resistance, breast cancer metastasis, FOXA1, GATA3, pleural effusions

## Abstract

Estrogen receptor‐alpha (ERα)‐positive breast cancer is often treated with antihormonal regimens. However, resistance to treatment is common, leading to metastatic disease. ERα activity requires the functional involvement of pioneer factors FOXA1 and GATA3, which enable ERα–chromatin binding and are crucial for ERα‐driven cell proliferation. FOXA1 was found increased in metastatic breast cancers in relation to the primary tumor, but a comprehensive clinical assessment thereof, in relation to different metastatic sites and endocrine therapy usage, is currently lacking. Prior cell line‐based reports, however, have revealed that FOXA1 is required for tamoxifen‐resistant tumor cell proliferation. We studied expression levels of ERα, GATA3, and FOXA1 by immunohistochemistry in samples from both primary tumors and various metastatic sites. For all factors, expression levels varied between the metastatic sites. For pleural metastases, strong variation was found in FOXA1 and GATA3 levels. Although GATA3 levels remained unaltered between primary breast cancer and pleural metastases, FOXA1 levels were reduced exclusively in metastases of patients who received endocrine therapies in the adjuvant setting, even though ERα was still expressed. Importantly, decreased FOXA1 levels in pleural metastases correlated with hormone irresponsiveness in the palliative setting, while no such correlation was found for GATA3. With this, we show divergent clinical correlations of the two ERα pioneer factors FOXA1 and GATA3 in metastatic breast cancer, where endocrine therapy resistance was associated with decreased FOXA1 levels in pleural metastases.

**Abbreviations**
CI: confidence interval
ERα: estrogen receptor‐alpha
ESR1: estrogen receptor 1
FFPE: formalin‐fixed paraffin‐embedded
FOXA1: forkhead box protein A1
GATA3: GATA binding protein 3
HR: hazard ratio
IHC: immunohistochemistry
TMA: tissue microarray

## 1 Introduction

Breast cancer is the most common malignancy in women with 1.7 million newly diagnosed cases annually worldwide and over 550 000 patients succumbing to the consequences of the disease each year (Ferlay *et al*., 2015). Around 70% of all breast tumors are of the luminal subtype, in which estrogen receptor‐alpha (ERα) is the main driver of cell proliferation, and consequently the prime drug target in treatment (Beelen *et al*., 2012; Droog *et al*., 2013). These patients are generally treated with endocrine therapies in the adjuvant setting: tamoxifen or aromatase inhibitors. Both types of drugs prevent ERα‐driven gene transcription, subsequently blocking tumor cell proliferation and tumor progression.

Despite an impressive reduction in recurrence risk, relapses do occur after adjuvant endocrine therapy (Early Breast Cancer Trialists’ Collaborative Group (EBCTCG) *et al*., 2011). These relapses may be due to either intrinsic or acquired drug resistance (Dalmau *et al*., 2014). Several intrinsic resistance mechanisms to hormonal intervention have been described, including activation of kinase pathways (Kok *et al*., 2011; de Leeuw *et al*., 2013; Zwart *et al*., 2007a) and overexpression of coregulators that are involved in ERα function (Zwart *et al*., 2007b, 2009). All these resistance mechanisms enable tumor cell proliferation despite hormonal therapy, ultimately giving rise to outgrowth of metastatic breast cancer (Kennecke *et al*., 2010).

Over the last years, acquired resistance to therapy has gained scientific attention. As a proportion of metastatic cancer cells of ERα‐positive breast cancer are likely to have evolved to proliferate and survive under endocrine therapy, it may not seem surprising that these resistance mechanisms can strongly differ from those responsible for intrinsic treatment resistance (Dalmau *et al*., 2014). Recently, several acquired resistance mechanisms have been identified, including activating mutations within the *ESR1* gene (around 20% of cases) (Robinson *et al*., 2013; Toy *et al*., 2013), genomewide reprogramming of the chromatin landscape (Magnani *et al*., 2013), and loss of ERα expression in metastases (termed receptor conversion; 6–25% of cases) (Hoefnagel *et al*., 2010; Van Poznak *et al*., 2015). Cumulatively, the currently known (genomic) aberrations only explain about 40–60% of endocrine‐resistant cases in the metastatic setting, and other mechanisms of acquired resistance to hormonal therapies are likely to exist. FOXA1 and GATA3, both well recognized as luminal breast cancer‐defining genes (Perou *et al*., 2000), play crucial roles in genomic functions of ERα. FOXA1 is required for ERα–chromatin interactions by rendering the chromatin accessible at designated ERα binding sites, in both hormone‐responsive and tamoxifen‐resistant cells (Hurtado *et al*., 2011). GATA3 facilitates FOXA1–chromatin interactions and directly affects chromatin loops that involve ERα (Theodorou *et al*., 2013). Jointly, FOXA1 and GATA3 are essential and sufficient to enable ERα–chromatin interactions, responsive gene activation, and cell proliferation (Kong *et al*., 2011).

FOXA1 expression was found increased in metastases in relation to primary breast cancer (Ross‐Innes *et al*., 2012). However, it remains to be determined whether levels of FOXA1 and GATA3 in metastatic breast cancer are affected by adjuvant endocrine therapy. We therefore immunohistochemically stained ERα, FOXA1, and GATA3 in metastatic breast cancer specimens from various sites. To find an explanation for the large variation in FOXA1 and GATA3 levels in samples of pleural metastases, we combined immunohistochemistry (IHC) data with prior treatment history in the adjuvant setting. We also combined these data with endocrine treatment response in the palliative setting.

Even though GATA3 levels were not affected in the metastases after adjuvant endocrine therapy, FOXA1 levels were selectively decreased in pleural metastases that arose after adjuvant treatment with endocrine therapeutics. Furthermore, patients with decreased FOXA1 levels were less responsive to endocrine therapeutics in the metastatic setting. These results suggest metastatic site‐selective variations of two well‐known ERα pioneer factors: GATA3 and FOXA1. Decreased FOXA1 levels in ERα‐positive pleural metastases are exclusively observed after prior adjuvant endocrine exposure, linking diminished FOXA1 levels with hormone irresponsiveness and endocrine therapy resistance in this setting.

## 2 Materials and methods

### 2.1 Patient material

A total of 210 individual ER‐positive, distant metastases of female patients with breast cancer were collected prospectively (pleural effusions only; *n* = 21) and retrospectively (both solid metastases and pleural effusion specimens; *n* = 189; Table 1). When available, paired formalin‐fixed paraffin‐embedded (FFPE) material of the primary breast tumor was collected as well (*n* = 81). The retrospectively obtained specimens (FFPEs) were collected in hospitals across The Netherlands: the Groene Hart Hospital Gouda, the Onze Lieve Vrouwe Gasthuis (Amsterdam), the Orbis Medical Center (Sittard), the Medical Center Leeuwarden, the University Medical Center St. Radboud (Nijmegen), the Meander Medical Center (Amersfoort), the Atrium Medical Center (Heerlen), the VieCuri Medical Center (Venlo), the Leiden University Medical Center, Bronovo Hospital (The Hague), Canisius Wilhelmina Hospital, Rijnstate Hospital (Arnhem), Diakonessenhuis (Utrecht), Isala Clinics (Zwolle), St. Franciscus Gasthuis (Rotterdam), Amphia Hospital (Breda), the Netherlands Cancer Institute (Amsterdam), and the University Medical Center Utrecht (described in Hoefnagel *et al*., 2010, 2012). Original diagnoses were made between 1988 and 2013.

**Table 1:** Clinicopathological characteristics of paired primary tumors and distant metastases

Feature	Grouping	*N* or value	%
Age at diagnosis of primary tumor (*n* = 81)	Mean	52	
	Range	27–83	
Tumor size in cm (*n* = 81)	Mean	3.1	
	Range	0.2–15	
	Unknown	22	
Histological grade	I	4	5
	II	26	32
	III	31	38
	Unknown	20	25
PR‐status primary tumor (*n* = 81)	Positive	71	88
	Negative	10	12
	Unknown	0	0
HER2‐status primary tumor (*n* = 81)	Positive	19	23
	Negative	62	77
	Unknown	0	0
Lymph node status (*n* = 81)	Positive	31	38
	Negative	18	22
	Unknown	32	40
Time between primary tumor and metastasis in days			
Total (*n* = 81)	Mean	373	
	Range	0–2839	
Solid metastases (*n* = 58)	Mean	152	
	Range	28–459	
Pleural effusions (*n* = 23)	Mean	446	
	Range	0–2839	
Location of metastasis (*n* = 210)	Liver	2	1
	Lung	7	3
	Brain	18	9
	Skin	23	11
	Bone	4	2
	Uterus/ovary	4	2
	Pleural effusion	152	72
Adjuvant therapy (*n* = 210)			
Endocrine therapy	Yes	72	34
	No	63	30
	Unknown	75	36
Chemotherapy	Yes	69	33
	No	53	25
	Unknown	88	42

### 2.2 Prospective pleural tumor cell collection and isolation

Fluid isolated from the pleural cavity was collected directly after drainage from patients at the University Medical Center Utrecht and the Netherlands Cancer Institute in Amsterdam. The cells in the pleural effusions were isolated by centrifugation, the erythrocytes were lysed (erythrocyte lysis buffer [pH 7.4]: 75 mm NH4Cl, 5 mm KHCO3, 400 μL 500 mm EDTA, and 500 mL ddH2O), and the remaining cells were either formalin‐fixed and paraffin‐embedded (Cellient; Hologic, 40180I10D0), or cryo‐stored at −80 °C (DMSO). Original diagnoses were made between 2014 and 2017.

### 2.3 Tissue processing

Immunohistochemical staining was performed on both pleural effusion samples and tissue microarrays (TMAs) (Jiwa *et al*., 2014) from 97 primary tumors and paired solid metastases. Three core biopsies (0.6 mm in diameter) of histologically representative regions of each tumor were used to construct the TMAs. For each FFPE sample, hematoxylin–eosin‐stained slides of the paraffin blocks were reviewed by an experienced breast pathologist (PJvD) to confirm the presence of malignancy. For the pleural effusions, only samples containing at least 20 tumor cells were selected. Ber‐EP4 monoclonal antibody staining (Epcam, 1 : 40, CCI24, DAKO) that labels epithelial tissues, but does not react with mesothelial cells, was used to confirm presence and quantity of tumor cells in the effusions. Due to loss of cores or not enough tumor cells left in the cores, 81/97 cases could be used for the final analyses.

This study was performed in accordance with the institutional medical ethical guidelines. The use of anonymous or coded leftover material for scientific purposes is part of the standard treatment agreement with patients, and therefore, informed consent was not required according to Dutch law (van Diest, 2002) for patients not actively opting out.

### 2.4 Immunohistochemical analyses

Sections of 4 μm were cut from the FFPE blocks of the pleural effusions, their primary breast tumors (if available), and the TMAs. IHC was performed with the Ventana autostainer (Roche, Tucson, Arizona, USA) according to the manufacturer's instructions. Mouse monoclonal antibodies used were against ERα (RTU, SP1; Roche, Tucson, USA), FOXA1 (1 : 100 000, WMAB‐2F83, Seven Hills Bioreagents), GATA3 (1 : 300, 5852 Cell Signaling Technology, Bioke), and Ber‐EP4 (Epcam, 1:40, CCI24, DAKO). Appropriate controls were used throughout.

Scoring of IHC slides was performed by two observers (PJvD and WAMES), who were blinded to clinicopathologic and molecular data. The percentage of positively stained nuclei was estimated side by side with the Ber‐EP4‐stained slide as a reference. For the TMAs, the mean score of all three cores was used as the final score per tumor.

### 2.5 Statistical analyses

IHC for ERα, FOXA1, and GATA3 positivity was assessed with a 1% threshold for positivity (ASCO guidelines) (Hammond *et al*., 2010), regardless of staining intensity. Percentages of nuclei expressing ERα, FOXA1, and GATA3 in primary tumors and their metastases in pleural effusions and solid tissues were compared by Wilcoxon signed rank test. Differences between hormonal treatment regimens were determined by Mann–Whitney U‐test and correlations between FOXA1 and GATA3 loss with Spearman's rho. Dichotomization for FOXA1 and GATA3 expression in treated and untreated patients was performed with ROC curves and compared with Fisher's exact test. Progression during treatment after first pleural effusion was visualized using Kaplan–Meier survival plots. Univariate and multivariate Cox proportional hazard regression analyses were performed to calculate hazard ratios (HR). All statistical analyses were performed using IBM SPSS Statistics version 21 and GraphPad Prism 6 (GraphPad Software, USA).

## 3 Results

### 3.1 Correlations of ERα, FOXA1, and GATA3 in primary and metastatic breast cancers

**Figure 1:**
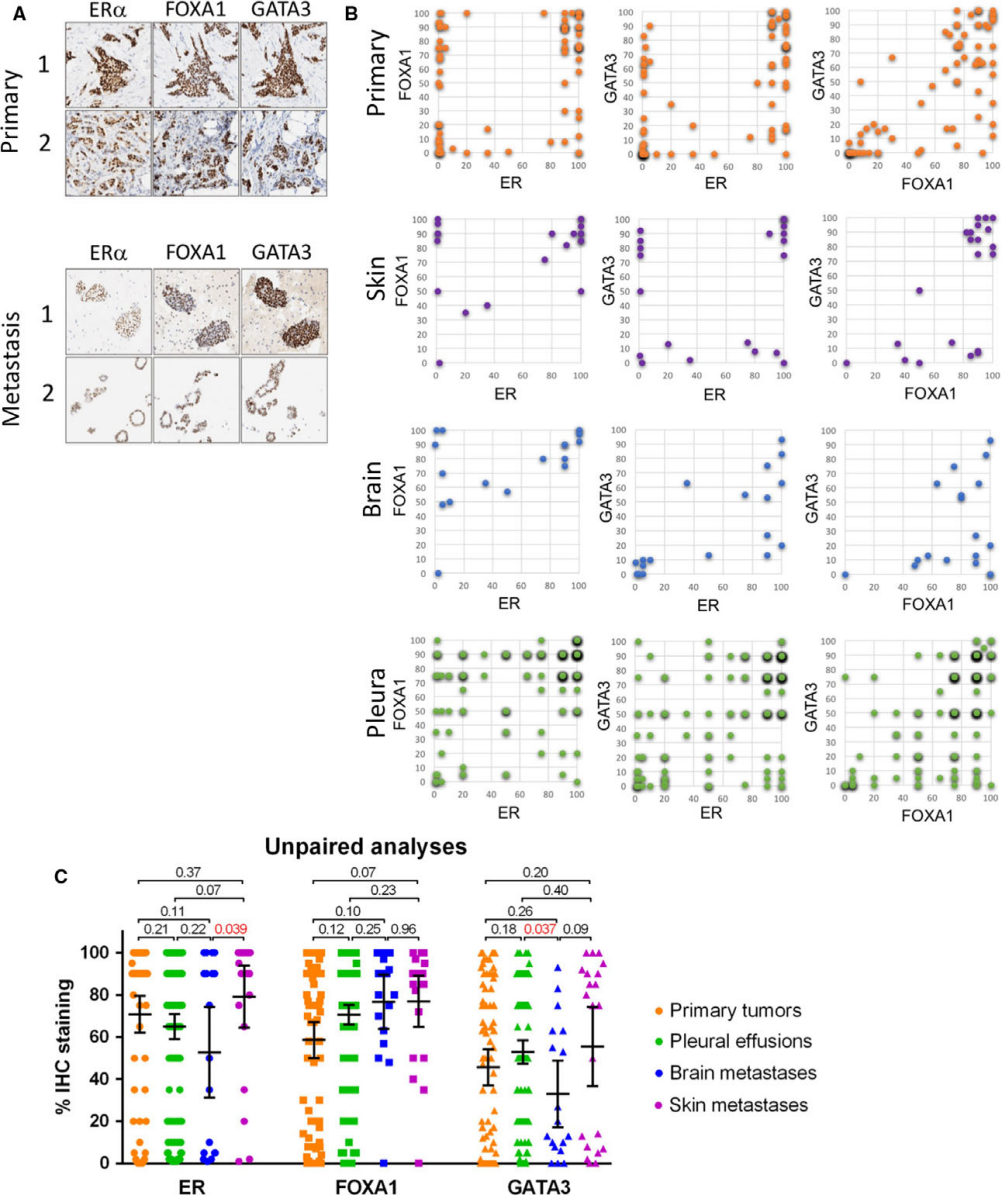
Loss of FOXA1 and GATA3 expression in ERα‐positive metastatic breast cancer. (A) Immunohistochemical analyses of ERα, FOXA1, and GATA3 in primary breast tumors and matched metastatic breast tumor cells. (B) Scatterplot visualizing immunohistochemical staining percentage of ERα vs. FOXA1, ERα vs. GATA3, and FOXA1 vs. GATA3 in primary breast cancer (orange) and skin (purple), brain (blue), and pleural effusion metastases (green). (C) Quantification of IHC staining for ERα, FOXA1, and GATA3 in primary breast cancers and metastases to the skin, brain, and pleural cavity using Wilcoxon signed rank test. Error bars indicate 95% confidence interval.

We gained access to 210 ERα‐positive (> 1% nuclear staining) metastatic breast cancer samples, with an enrichment for pleural effusion metastases (*n* = 152). In addition, two liver, seven lung, eighteen brain, twenty‐three skin, four bone, and four uterus or ovary metastases were included. For 39% (81/210) of these samples, paired primary breast cancers could be retrieved from the pathology registry (for clinicopathological variables, Table 1). As expected for transcription factors, clear nuclear signal intensity was observed for ERα, FOXA1, and GATA3 in the primary breast cancers as well as the metastatic samples (Fig. 1A). Out of 135 patients for whom treatment history was present, 72 patients received adjuvant endocrine therapy (53%). As expected and in line with literature (Perou *et al*., 2000), most ERα‐positive primary breast cancers we studied also expressed FOXA1 (*n* = 73/81, 90%; staining percentage range 3–100%, mean 70%) and GATA3 (*n* = 63/81, 78%; staining percentage 1–100%, mean 58%). Significant correlations were found between levels of ERα and FOXA1 (ρ = 0.268; *P* = 0.018; Spearman's rho), ERα and GATA3 (ρ = 0.549; *P* < 0.001; Spearman's rho), and GATA3 and FOXA1 (ρ = 0.707; *P* < 0.001; Spearman's rho) in the primary breast tumors and in the metastatic setting (ρ = 0.349, *P* < 0.001; ρ = 0.459, *P* < 0.001; ρ = 0.535, *P* < 0.001, respectively; Spearman's rho). Qualitatively, we observed a bimodal distribution in the primary samples for ERα, FOXA1, and GATA3, separating high‐ and low‐percentage samples in two distinct groups (Fig. 1B; statistics are shown in Fig. 1C). These patterns differed between the different metastatic sites, with FOXA1 highly expressed in practically all skin and brain metastases, while GATA3 more often showed lower expression levels in brain metastases. For pleural effusion samples, a different pattern was found for all factors, displaying a large variation in signal intensity of ERα, FOXA1, and GATA3 between samples, occupying the full dynamic range between 0 and 100% (Fig. 1B; statistics are shown in Fig. 1C).

Compared to levels of GATA3 and FOXA1 in primary tumors, average levels were similar in some metastatic sites, while expression differed in others (Fig. 1C). Jointly analyzing all metastases as one population, this trend was not significant for FOXA1 (*P* = 0.730; mean staining percentage primary 71% vs. metastasis 64%; Wilcoxon signed rank test), while for GATA3 it was (*P* = 0.036; mean staining percentage primary 59% vs. metastasis 38%; Wilcoxon signed rank test). In contrast to pleural effusions but in agreement with previous reports (Ross‐Innes *et al*., 2012; Tangen *et al*., 2014), FOXA1 levels were increased in solid metastases (*P* = 0.002; mean staining percentage primary 53% vs. metastasis 74%; Wilcoxon signed rank test; Fig. S3). This effect was most prominent in solid metastases to the skin (*P* = 0.070; Wilcoxon signed rank test; Fig. 1C). No difference in GATA3 level was found between primary breast cancer and solid metastases (*P* = 0.658; mean staining percentage primary 40% vs. metastasis 42%; Wilcoxon signed rank test; Fig. S3).

### 3.2 FOXA1 and GATA3 expression in relation to adjuvant endocrine therapy exposure

Even though FOXA1 and GATA3 levels have previously been studied in the context of primary and metastatic breast cancer, no thorough assessment has been performed to date in relation to prior endocrine therapy usage.

We chose to focus on pleural effusion metastases (*n* = 152), due to the large interpatient variation observed in levels of FOXA1 and GATA3 for this metastatic site. As *ESR1* activating hotspot mutations are reported enriched in endocrine‐resistant metastatic breast tumors (Toy *et al*., 2013), we tested for any of the helix 12 activating hotspot mutations in 11 randomly selected samples from adjuvant tamoxifen‐treated patients (Fig. S1). None of these samples exposed activating hotspot mutations. Therefore, it seems unlikely that activating ESR1 point mutations is a major contributor in our cohort of pleural metastases.

**Figure 2:**
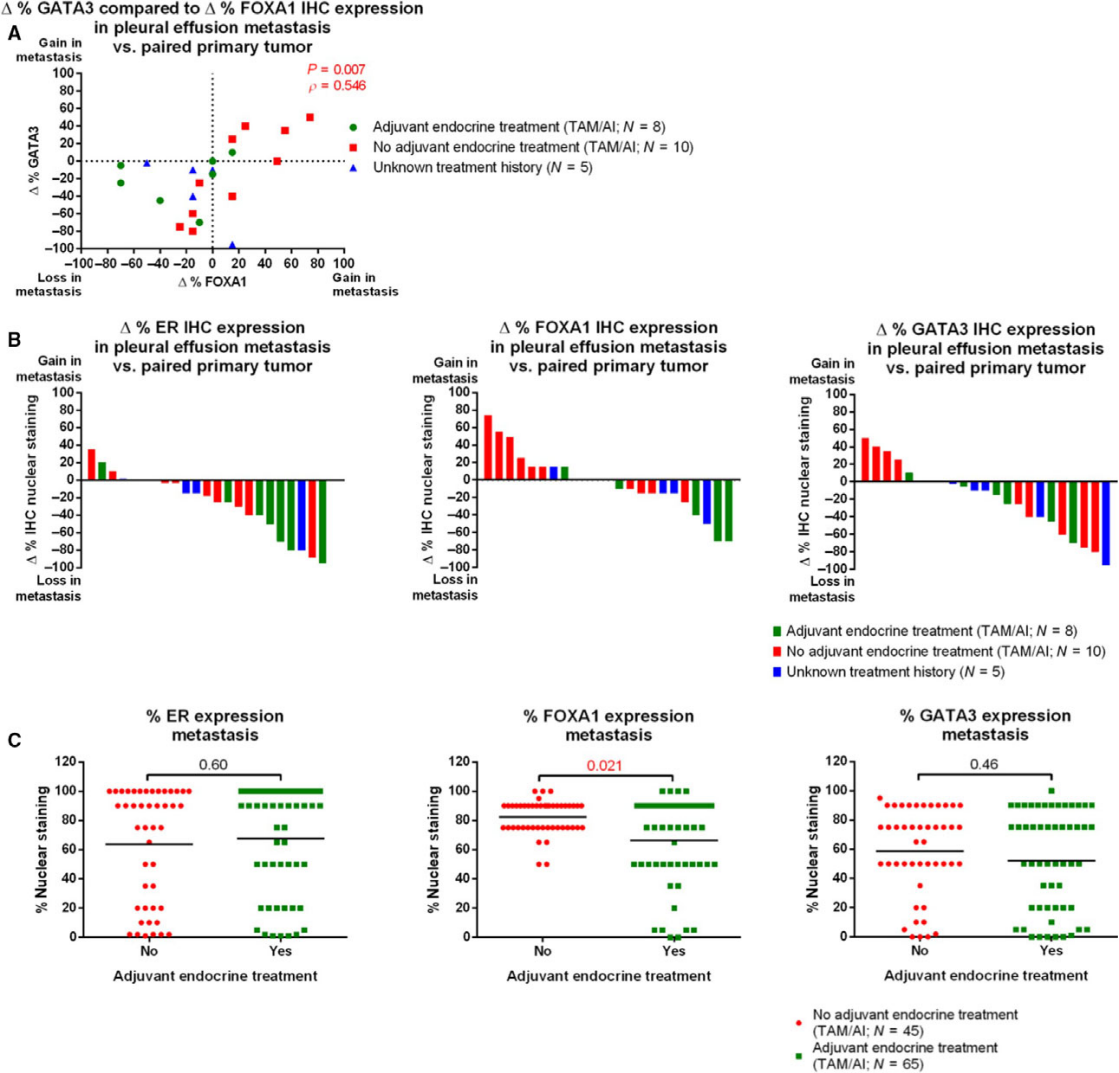
Decreased FOXA1 expression levels in metastases after prior tamoxifen exposure. (A) Scatterplot visualizing percentage change of FOXA1/GATA3 levels in paired pleural metastases vs. primary breast tumors from the same patients. Correlation was calculated with Spearman's rho. Samples from patients receiving adjuvant tamoxifen (green), no adjuvant endocrine treatment (red), or unknown adjuvant treatment (blue) are visualized separately. (B) Waterfall plot showing changed expression of ERα (left), FOXA1 (middle), and GATA3 (right) in paired analyses on pleural metastases relative to primary breast cancers, compared with Mann–Whitney U‐test. Patients receiving adjuvant tamoxifen (green), no adjuvant endocrine treatment (red), or unknown adjuvant treatment (blue) are indicated. (C) Expression levels of ERα (left), FOXA1 (middle), and GATA3 (right) in pleural metastases, from patients who either did (green) or did not (red) receive adjuvant endocrine treatment, compared with Mann–Whitney U‐test. Mean is indicated.

Changes in FOXA1 levels between primary tissue and paired pleural metastases typically co‐occurred with changes in GATA3 in the same samples (ρ = 0.546, *P* = 0.007; Spearman's rho; Fig. 2A). The same trend was encountered for primary tumors compared to pleural effusion and solid metastases (ρ = 0.458, *P* = 0.001; Spearman's rho; Fig. S4). Patients that had received adjuvant endocrine therapy showed a trend toward a greater decrease (Δ%) of FOXA1 expression levels in pleural metastases compared to the primary tumor vs. patients that had not received adjuvant therapy (*P* = 0.085; Mann–Whitney U‐test; Fig. 2B), while this was not the case for GATA3 (*P* = 0.779; Mann–Whitney U‐test). Also in unpaired analyses, FOXA1 expression in pleural effusions was significantly lower in patients who received adjuvant endocrine therapy compared to the pleural samples of patients who did not receive any adjuvant endocrine treatment (*P* = 0.021; *n* = 100; Mann–Whitney U‐test; Fig. 2C). This effect was not seen for GATA3 (*P* = 0.46; *n* = 100; Mann–Whitney U‐test) or ERα (*P* = 0.60; *n* = 100; Mann–Whitney U‐test). Tamoxifen was the most dominant adjuvant endocrine treatment in this cohort (adjuvant tamoxifen *n* = 42, adjuvant aromatase inhibitor *n* = 4, combination of both *n* = 8), preventing sufficiently powered subanalysis based on type of endocrine therapy.

FOXA1 and GATA3 levels in solid metastases were not associated with adjuvant hormonal therapy, in both unpaired (*P* = 0.197 for FOXA1 and *P* = 0.568 for GATA3; Mann–Whitney U‐test) and paired analyses (untreated patients: *P* = 0.073 for FOXA1 and *P* = 0.638 for GATA3; patients receiving adjuvant hormonal therapy: *P* = 0.088 for FOXA1 *P* = 0.296 for GATA3; Wilcoxon signed rank test; Fig. S2).

### 3.3 FOXA1 and GATA3 expression levels in relation to treatment response in the metastatic setting

**Figure 3:**
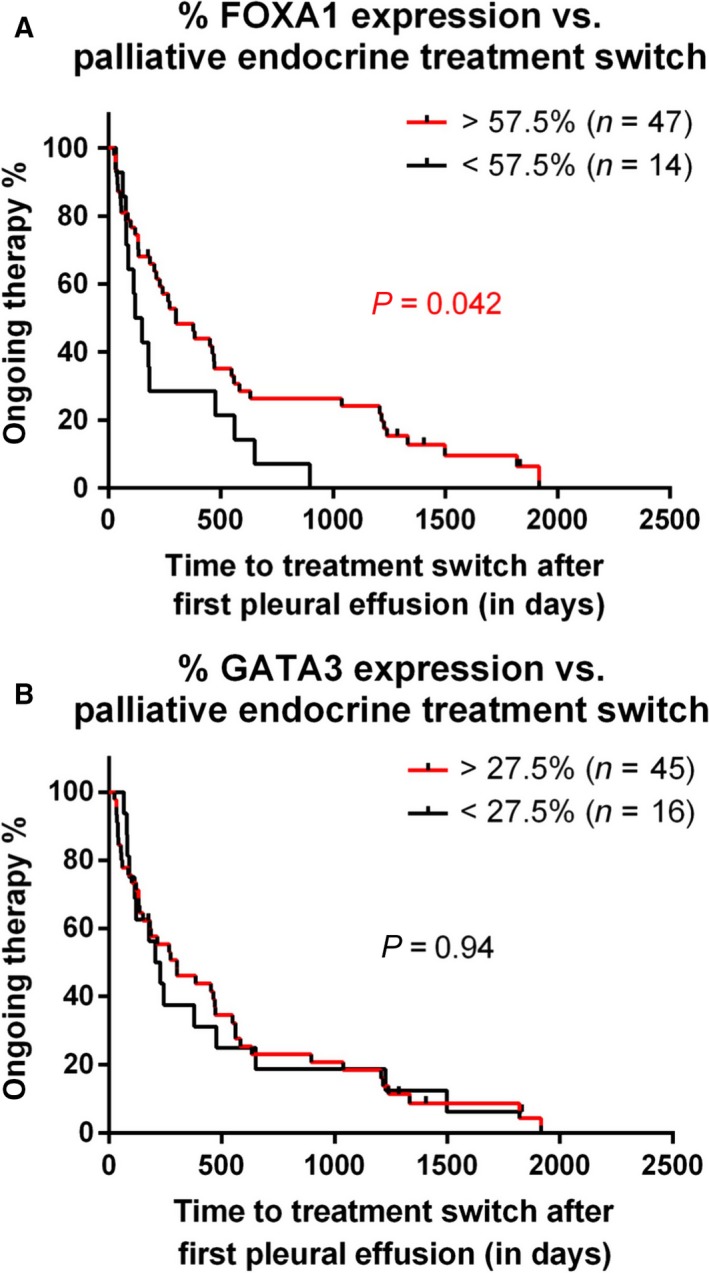
FOXA1 expression levels are associated with response to endocrine therapy in the metastatic setting. Kaplan–Meier plot indicating time to treatment switch after first pleural effusion, separately analyzing FOXA1 (top) and GATA3 (bottom), calculated with log‐rank test. Dichotomization for FOXA1 and GATA3 expression in treated and untreated patients was performed with ROC curves.

As FOXA1 and GATA3 are described as essential factors in ERα functioning (Hurtado *et al*., 2011; Theodorou *et al*., 2013), decreased levels of either of these two factors would be associated with nonfunctional ERα and would imply resistance to endocrine therapeutics in this setting. This was tested in all pleural metastatic samples of patients receiving endocrine treatment for metastatic disease, in which time on first endocrine therapy after pleural effusion before switching to another type of treatment (mostly due to disease progression) was used as endpoint. FOXA1 decrease was significantly associated with a shorter time on first endocrine therapy (Fig. 3A; *P* = 0.042; HR = 0.463; 95% CI: 0.220–0.973; log‐rank test; cutoff for positivity determined with ROC curves). For GATA3, this effect was not observed (Fig. 3B; *P* = 0.937; HR = 0.976; 95% CI: 0.536–1.776; log‐rank test). To adjust for possible confounders, univariate Cox proportional hazard regression analysis was performed on traditional predictive clinicopathological factors: disease‐free interval, age at diagnosis of primary tumor, tumor size, immunohistochemical markers (PR and HER2), and histological grade and type (Table S1). Only disease‐free interval and histological type showed significant association with time to treatment switch (Table 2). When put in a multivariate model, a shorter disease‐free interval was correlated with a longer time to treatment switch (HR: 2.653, 95% CI: 1.074–6.552, *P* = 0.034), independent of FOXA1 and GATA3 decrease.

**Table 2:** Univariate Cox proportional hazard regression analysis of time to treatment switch after first pleural effusion (*n* = 61)

Category	*N*	HR	95% CI	*P*
Disease‐free interval				
<5 years	13	2.541	1.074–6.012	0.034
> 5 years	16	1		
Unknown	32			
Age at diagnosis				
<55 years	18	1		
≥ 55 years	16	1.238	0.592–2.590	0.571
Unknown	27			
Type				
Ductal	31	1		
Lobular	4	0.284	0.090–0.899	0.032
Unknown	26			
Stage				
I/II	18	1		
III	6	0.532	0.176–1.607	0.263
Unknown	37			
Size				
≤ 2 cm	13	0.587	0.275–1.254	0.169
> 2 cm	17	1		
Unknown	31			
PR (IHC) primary tumor				
<1%	7	0.760	0.305–1.895	0.557
≥ 1%	18	1		
Unknown	36			
HER2 (IHC) primary tumor				
0/1 + /2 +	23	1		
3 +	2	0.699	0.156–3.138	0.640
Unknown	36			

Cumulatively, these data illustrate that FOXA1 expression is decreased in progressive metastatic breast cancer to the pleural cavity. This decrease in FOXA1 levels in metastases is discordant with previous reports in solid metastases (Ross‐Innes *et al*., 2012), suggesting organotropism of FOXA1 levels in metastatic breast cancer. Furthermore, FOXA1 levels were decreased exclusively for the patients who received endocrine therapy in the adjuvant setting, and were indicative for resistance to endocrine intervention in metastatic disease.

## 4 Discussion

Years of continuous drug exposure in the adjuvant setting provides a massive selection pressure on the tumor cells for evading the blocking effects of endocrine treatment (Robinson *et al*., 2013; Toy *et al*., 2013). Recently, several reports identified a distinct set of activating point mutations within the ERα trans‐activating domain, only found in ~ 20–40% of metastases with acquired endocrine therapy resistance (Jeselsohn *et al*., 2017; Robinson *et al*., 2013; Toy *et al*., 2013). Also, epigenetic reprogramming of the chromatin landscape can underlie endocrine therapy‐resistant breast cancer by switching from ERα to NOTCH signaling (Magnani *et al*., 2013). In another 6–25% of patients, ERα expression is lost in metastases while the primary tumor was ERα‐positive (Van Poznak *et al*., 2015). Still, these known mechanisms of endocrine therapy resistance recapitulate only about 40–60% of all observed resistance, leaving many endocrine therapy unresponsive cases unexplained. In the current study, we position decreased levels of FOXA1 as a potential novel mechanism of acquired endocrine therapy resistance, which could be further developed as a biomarker.

GATA3 and FOXA1 are both essential transcription factors for ERα action, enabling its chromatin interactions and consequently facilitating ERα‐responsive gene activity. As such, both GATA3 and FOXA1 are termed key luminal breast cancer‐defining genes and are expressed in virtually all ERα‐positive primary breast cancers (Perou *et al*., 2000). In pleural effusion metastases, however, FOXA1 expression is decreased under evolutionary selection pressure of continuous ERα blockade, while GATA3 levels were not affected.

Persistent and increased FOXA1 expression was seen in solid metastases that arose from an ERα‐positive breast cancer, regardless of the sites of metastasis (Ross‐Innes *et al*., 2012). This is consistent with our data, where we find FOXA1 generally increased relative to the primary tumor in metastases to the skin and brain. However, in pleural metastases no increase in FOXA1 levels was observed relative to the primary tumor, implicating metastatic site organotropism in relation to FOXA1 levels. In line with this concept, we did not observe any of the previously identified hotspot *ESR1* mutations, found in 20–40% of metastatic breast cancers that progressed after adjuvant endocrine therapy (Robinson *et al*., 2013; Toy *et al*., 2013). Possibly, malignant pleural effusions are less enriched for *ESR1* mutations as compared to solid metastases. However, our cohort is enriched for cases who received tamoxifen in the adjuvant setting, and activating ESR1 mutations are mostly identified in patients who previously received aromatase inhibitors and rarely observed in patients solely receiving tamoxifen (Reinert *et al*., 2017), indicating that our study is possibly underpowered for stronger conclusions.

In about 20% of malignant pleural effusions that arose after adjuvant endocrine therapy, ERα expression is lost (Van Poznak *et al*., 2015), and endocrine therapy will not be effective. We hypothesize that decreased FOXA1 levels in metastases that developed under selection pressure of continued endocrine therapeutics, results in a comparable disruption of the ERα signaling axis and loss of sensitivity to endocrine therapies. Reports that describe increased FOXA1 levels in solid metastases limited the analyses in relation to the primary tumor, without incorporating treatment information (Ross‐Innes *et al*., 2012). We now show that adjuvant endocrine therapy associates with decreased FOXA1 levels in pleural metastases.

Several guidelines advise to reassess ERα status in metastases by immunohistochemistry (Carlson *et al*., 2012; Hammond *et al*., 2010). This may identify potential loss of ERα expression, but does not provide any information on ERα functionality in case ERα expression is maintained. As ERα expression alone is not per definition informative about its functional activity, additional biomarkers may aid in identifying ERα functionality. We show here that decreased FOXA1 expression is associated with poor outcome after endocrine therapy for pleural metastases, even though these results do require confirmation in a second cohort. Most importantly, validation of these findings in an independent case–control study or randomized trial of endocrine therapy vs. nil (or another anticancer therapy not targeting the ER) would be required to assess whether FOXA1 levels are a prognostic factor or whether they are predictive for endocrine therapy response in the metastatic setting.

This study design enabled us to study differences in immunohistochemical markers between primary tumors and metastases by paired analyses. To our knowledge, this is the largest sample set in which influence of endocrine therapy is investigated on FOXA1 and GATA3 expression in paired primary tumors and metastases. However, the influence of tumor heterogeneity should not be overlooked. As we used patient samples that also were used for diagnostic purposes, only parts of the tumors were available for our analyses. Also, treatment history was not present for all patients. Furthermore, we used immunohistochemical staining percentages to compare ERα, FOXA1, and GATA3 in primary tumors vs. metastases, and as this is a semiquantitative approach, it would be very valuable to better quantify these findings with other molecular techniques in further validation studies. We deliberately chose to limit data analyses and interpretation on the most robust variable in this setting, being percentage of positive nuclei, as reproducibility for scoring intensity of FOXA1 and GATA3 immunohistochemistry appeared to be lower compared to percentage of positive nuclei. Further, FOXA1 intensity turned out to be affected by fixation time, while percentage of staining was not. Finally, this data set was largely enriched for pleural effusion metastases, preventing us to draw strong conclusions on solid metastases. Recently, we already showed that steroid hormone receptor expression can differ largely between primary tumors and solid and effusion metastases in the same patient (Schrijver *et al*., 2017). Furthermore, as it is currently unknown to what degree the hormonal levels are comparable between different solid metastases and pleural effusions, different evolutionary selection pressure may occur between these different metastatic sites.

As around 11% of patients with breast cancer eventually present with symptomatic pleural effusions and at autopsy 36–65% of patients retrospectively suffered from this condition (American Thoracic Society, 2000; Apffelstaedt *et al*., 1995), future studies should validate our findings in other cohorts and may reveal predictive biomarkers for endocrine therapy sensitivity in pleural metastases, facilitating tailored treatment selection for this large group of patients with metastatic disease.

## 5 Conclusions

Immunohistochemical ERα, GATA3, and FOXA1 expression levels vary between primary breast tumors and paired metastases. FOXA1 is reduced in pleural metastases of patients who received endocrine therapies in the adjuvant setting. Decreased FOXA1 levels are associated with poor outcome after endocrine therapy in the metastatic setting.

## Author contributions

WS and KS performed sample collection and processing. Prospective sample collection was performed and/or facilitated by WS, KS, WZ, MvdH, CJ, SS, and RH. Data analyses were executed by WS, KS, MD, and AvR. Pathological assessment of slides was performed by PvD and JW. WS, CM, SCL, PvD, and WZ wrote the article, with the input of all other authors.

## Supporting information


**Fig. S1.** ESR1 helix 12 activating hotspot mutations in 11 randomly selected pleural effusion samples from adjuvant tamoxifen‐treated patients, as analyzed by Sanger sequencing.
**Fig. S2.** Expression levels of ERα, FOXA1, and GATA3 in pleural metastases compared to paired primary tumors, from patients who either did or did not receive adjuvant endocrine treatment.
**Fig. S3.** Quantification of IHC staining for ERα, FOXA1, and GATA3 in primary breast cancers and solid metastases.
**Fig. S4.** Scatterplot visualizing percentage change of FOXA1/GATA3 levels in paired solid metastases vs. primary breast tumors from the same patients.

**Table S1.** Clinicopathological characteristics of patient samples used for the Kaplan–Meier plot in Fig. 3 and univariate Cox proportional hazard regression analysis.
