# Supplementary material for: FOXA1 levels are decreased in pleural breast cancer metastases after adjuvant endocrine therapy, and this is associated with poor outcome
Source: Mol Oncol. 2018 Oct 12;12(11):1884–94. doi: 10.1002/1878-0261.12353 (PMC6210032; doi:10.1002/1878-0261.12353)
Supplement: Supplementary file 1 — Fig. S1. ESR1 helix 12 activating hotspot mutations in 11 randomly selected pleural effusion samples from adjuvant tamoxifen‐treated patients, as analyzed by Sanger sequencing. Fig. S2. Expression levels of ERα, FOXA1, and GATA3 in pleural metastases compared to paired primary tumors, from patients who either did or did not receive adjuvant endocrine treatment. Fig. S3. Quantification of IHC staining for ERα, FOXA1, and GATA3 in primary breast cancers and solid metastases. Fig. S4. Scatterplot visualizing percentage change of FOXA1/GATA3 levels in paired solid metastases vs. primary breast tumors from the same patients. [file MOL2-12-1884-s001.pdf]

S1

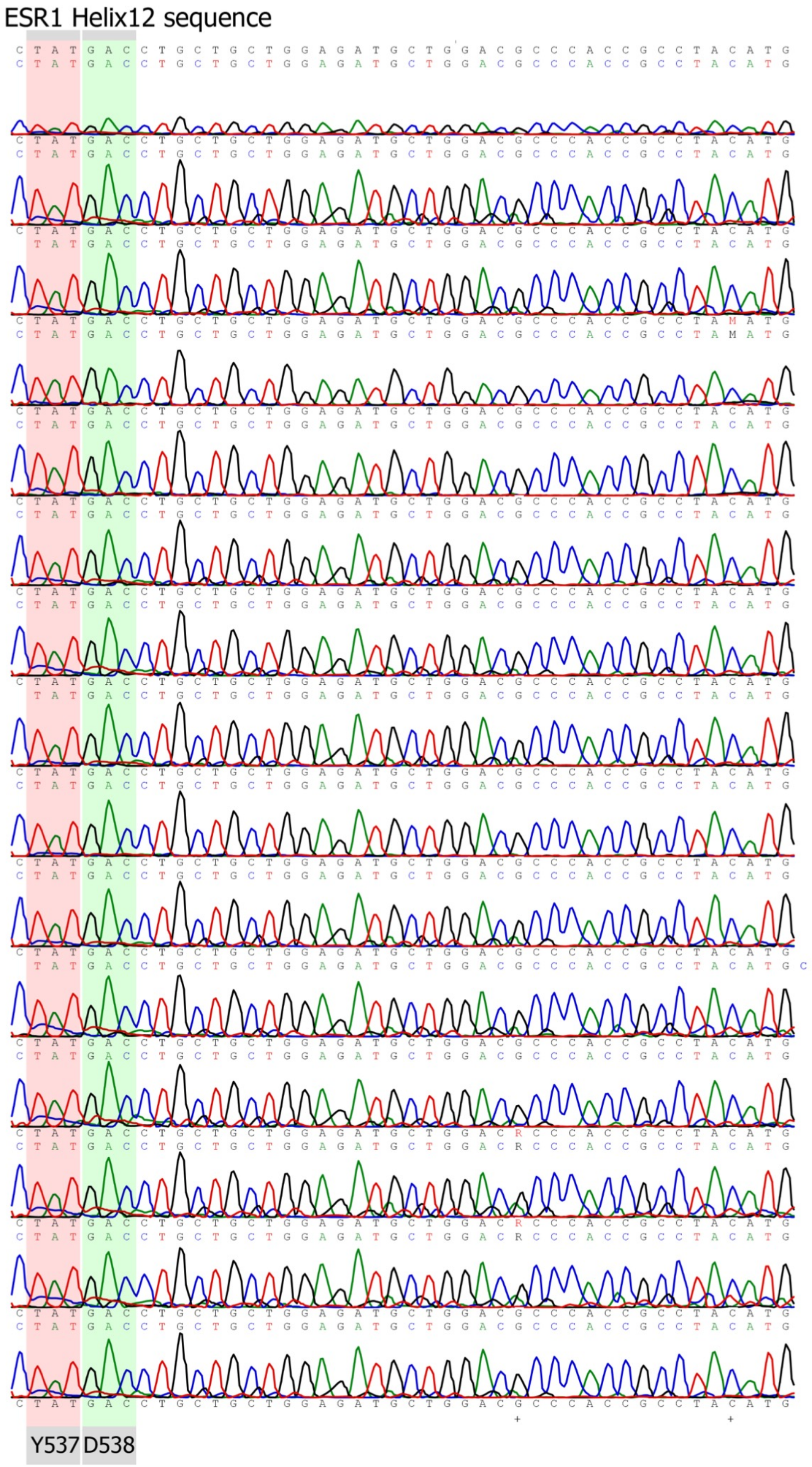

Supplementary Figure S1: ESR1 Helix 12 activating hotspot mutations in 11 randomly selected pleural effusion samples from adjuvant tamoxifen-treated patients, as analysed by Sanger sequencing.

S2

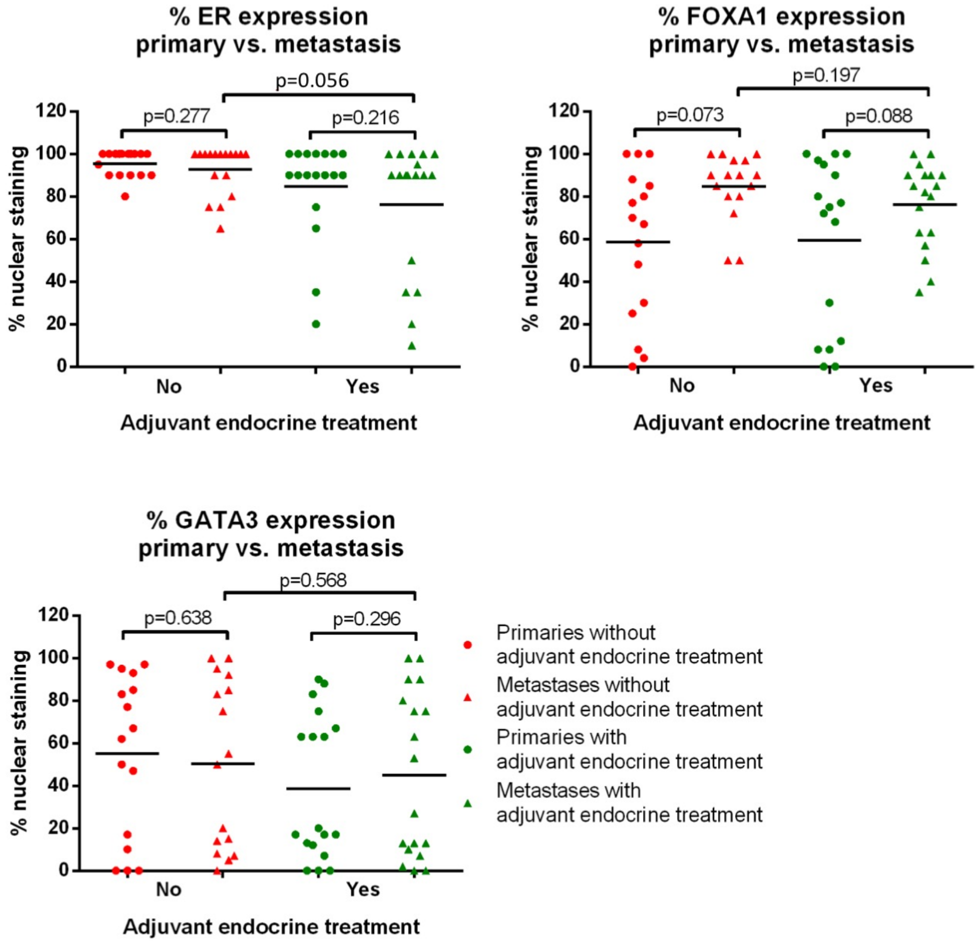

Supplementary Figure S2: Expression levels of ERα, FOXA1 and GATA3 in pleural metastases (triangle) compared to paired primary tumors (circle), from patients who either did (green) or did not (red) receive adjuvant endocrine treatment, compared with Wilcoxon signed rank test. Mean is indicated.

S3

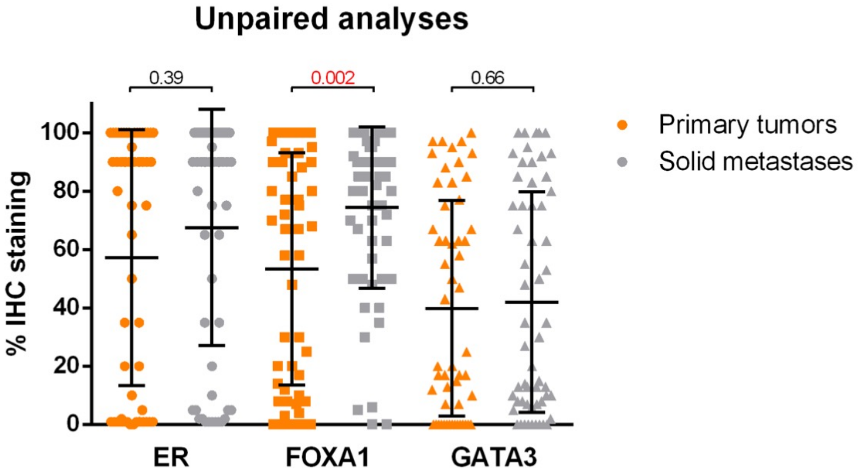

Supplementary Figure S3: Quantification of IHC staining for ERα, FOXA1 and GATA3 in primary breast cancers (orange) and solid metastases (grey) using Wilcoxon signed rank test. Error bars indicate 95% confidence interval.

S4

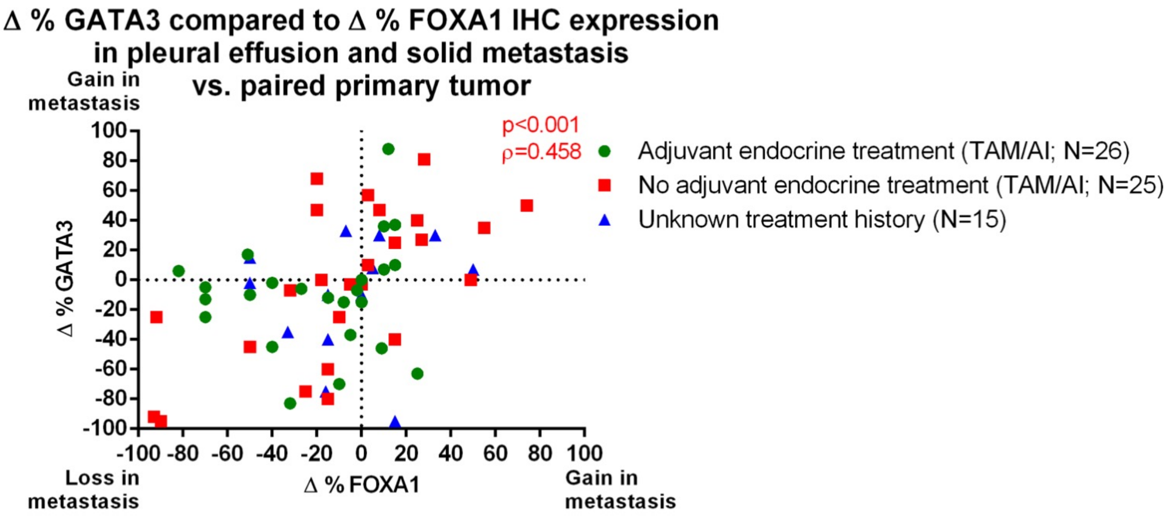

Supplementary Figure S4: Scatterplot visualizing percentage change of FOXA1/GATA3 levels in paired solid metastases versus primary breast tumors from the same patients. Correlation was calculated with Spearman's Rho. Samples from patients receiving adjuvant tamoxifen (green), no adjuvant endocrine treatment (red) or unknown adjuvant treatment (blue) are visualized separately.
