# Supplementary material for: FOXA1 levels are decreased in pleural breast cancer metastases after adjuvant endocrine therapy, and this is associated with poor outcome
Source: Mol Oncol. 2018 Oct 12;12(11):1884–94. doi: 10.1002/1878-0261.12353 (PMC6210032; doi:10.1002/1878-0261.12353)
Supplement: Supplementary file 3 [file MOL2-12-1884-s003.docx]

**Supplementary Figure legends**

**Supplementary Figure S1:** Sanger sequencing analysis on ERα ligand-binding domain hotspot mutations in metastatic breast cancer specimens.

**Supplementary Figure S2:** Expression levels of ERα, FOXA1 and GATA3 primary breast cancers (circles) and solid metastases (triangles), from patients who either did (green) or did not (red) receive adjuvant endocrine treatment. Percentage of nuclear staining is shown.

**Supplementary Figure S3:** Quantification of IHC staining for ERα, FOXA1 and GATA3 in primary breast cancers and solid metastases, using Wilcoxon signed rank test.

**Supplementary Figure S4:** Scatterplot visualizing percentage change of FOXA1/GATA3 levels in paired solid and pleural metastases versus primary breast tumors from the same patients. Samples from patients receiving adjuvant tamoxifen (green), no adjuvant endocrine treatment (red) or known adjuvant treatment (blue) are visualized separately.
